# Development of curative therapies for sickle cell disease

**DOI:** 10.3389/fmed.2022.1055540

**Published:** 2022-11-24

**Authors:** Yvette C. Tanhehco, Ghazala Nathu, Ljiljana V. Vasovic

**Affiliations:** ^1^Department of Pathology and Cell Biology, Columbia University Irving Medical Center, New York, NY, United States; ^2^Department of Clinical Pathology, Bassett Healthcare Network—Cobleskill Regional Hospital, Cobleskill, NY, United States; ^3^Department of Pathology and Laboratory Medicine, Weill Cornell Medicine, New York, NY, United States

**Keywords:** Sickle Cell Disease (SCD), gene therapy, hematopoietic stem cell transplantation (HSCT), disease modifying drugs, lentiviral vectors (LVS)

## Abstract

Recent advances in managing Sickle Cell Disease (SCD) significantly improved patient survival and quality of life. Disease-modifying drug therapies such as hydroxyurea, L-glutamine, voxelotor, and crizanlizumab reduce pain crises and severe complications. Allogeneic hematopoietic stem cell transplantation using matched-sibling donors is currently the only standard curative option; however, only a small proportion of patients have such donors. Cord blood and haploidentical transplantation with a modified conditioning regimen have expanded the allogeneic donor pool, making the therapy available to more patients. Gene therapy is a promising cure that is currently undergoing clinical trials and different approaches have demonstrated efficacy. Multidisciplinary expertise is needed in developing the best treatment strategy for patients with SCD.

## Introduction

Sickle Cell Disease (SCD) is caused by a single point mutation in the hemoglobin beta gene that codes for a valine in the sixth amino acid position instead of the wild type glutamic acid creating hemoglobin S (HbS) (1). Under deoxygenated conditions, HbS polymerizes causing erythrocytes to assume a sickle shape (2). These HbS-containing erythrocytes are insufficient for transporting oxygen. The conformational change of erythrocytes and increased surface expression of adhesion molecules can lead to blood vessel occlusion, painful vaso-occlusive crises (VOC), and long-term damage to tissues and organs (3).

SCD is among the most prevalent inherited monogenic disorders worldwide. It affects Black Americans more than any other racial group. It is believed that more than 20 million people worldwide and 80,000–100,000 Americans battling SCD, with an average of 3,000 children being born with it annually in the United States (US) (4). Newborn screening programs, early interventions, and preventive care improved pediatric patients' survival. Nevertheless, the life span of patients with SCD is about 20 years shorter than the general population. The National Institutes of Health Cure Sickle Cell Initiative established a collaborative, patient-centric research program aimed at discovering effective genetic treatments for SCD. This is a systematic effort to support the development of curative therapies and empower understanding of basic research, clinical trials, and patient support which have wide implications for educational and community engagement activities (5).

Traditional therapies for SCD include pain and symptom management. In certain cases, physicians might recommend a blood transfusion in order to instill an appropriate amount of hemoglobin A containing erythrocytes, prevent strokes, and treat anemia (6, 7). Simple transfusion, chronic transfusion or red blood cell exchange protocols have been utilized. As with any transfusion, there is a risk for alloimmunization due to incomplete phenotypic matches, iron accumulation, and other transfusion-related adverse events.

The first disease modifying drug therapy that was used to reduce complications is hydroxyurea (Table 1). Subsequently, the US Food and Drug administration (FDA) approved L-glutamine, voxelotor, and crizanlizumab-tmca to reduce pain crises, sickling, and VOC which have all significantly improved the quality of life of patients with SCD (7).

**Table 1:** Sickle cell disease therapeutic options.

**Therapeutic options**	**Mechanism of action**
**Disease modifying drug therapies**
Hydroxyurea	Increase HbF
L-glutamine	Increase NAD and NADH, preventing VOD
Crizanlizumab	Monoclonal antibody to P-selectin that blocks the adhesion of activated erythrocytes, neutrophils and platelets, preventing VOD
Voxelotor	Increase oxygen affinity, stabilize oxygenated HbS, inhibit HbS polymerization
Complement inhibitors	Improvement in intravascular hemolysis, anemia, transfusion independence, and thrombotic events
**Allogeneic hematopoietic stem cell transplantation (HSCT)**
Matched related donor transplant	Establishing donor-derived erythropoiesis
Cord blood transplant	Establishing donor-derived erythropoiesis
Haploidentical familial donor transplant	Establishing donor-derived erythropoiesis
Alternate donor sources	Establishing donor-derived erythropoiesis
**Gene therapy/autologous HSCT**
Gene addition (Antisickling Gene)	Lentiviral vector with T87Q mutation (HbA^T87Q^), leading to steric inhibition of HbS polymerization
Gene editing (HbF Induction)	Upregulating HbF expression with CRISPR Cas-9 disruption of BCL11a gene

VOD, Vaso-occlusive crises; HSCT, hematopoietic stem cell transplantation.

Thus, far, the only curative approach to SCD has been a hematopoietic stem cell (HSC) transplantation. Allogeneic stem cells lacking the genetic mutation will yield healthy erythrocytes in the recipient (8). However, the potential complications of an allogenic transplant, such as rejection or graft-vs. host disease (GVHD), are considerable potentially causing morbidity and death. The major hurdle of procuring a fully matched HLA donor can be circumvented by using cord blood and haploidentical donors.

There is a new treatment paradigm on the horizon that aims to cure SCD by correcting the core issue (i.e., the genetic mutation) through gene therapy. Gene therapy approaches are variable and may involve introducing a new gene into the patient to be integrated and expressed or to modify the native gene itself through gene editing. All of these methodologies require that the HSCs, the targets of these therapies, be collected and harvested from the patient. Genetic modification then ensues *in-vitro* followed by transplantation back to the patient after the residual marrow population is ablated using chemotherapy (9).

This minireview discusses the available therapeutic modalities for SCD including gene therapy.

## Disease modifying drug therapies

### Hydroxyurea

Hydroxyurea (HU) is a disease modifying drug (DMD) therapy that was approved by the US FDA in 1998 for the treatment of adults (>18 years) with SCD who have had at least 3 painful crises in the past year (10). HU increases the level of fetal hemoglobin (HbF) and reduces the tendency for HbS to polymerize, preventing RBCs from sickling and causing VOC (11). HU also reduces circulating leukocytes and inflammatory mediators. A double-blind, randomized clinical trial enrolled 148 men and 151 women at least 18 years of age with sickle cell anemia at 21 clinics. Study subjects must have had at least 3 crises in the year before entry into the study. Study results showed that the 152 patients who received HU treatment had lower annual rates of crises than the 147 patients who received placebo (median, 2.5 vs. 4.5 crises per year, *P* < 0.001). The median times to the first crisis (3.0 vs. 1.5 months, *P* = 0.01) and the second crisis (8.8 vs. 4.6 months, *P* < 0.001) were longer with HU treatment. Fewer patients who were treated with HU had chest syndrome (25 vs. 51, *P* < 0.001), and fewer received transfusions (48 vs. 73, *P* = 0.001). No significant adverse effects were observed but its use must be carefully monitored. The beneficial effects of HU did not manifest for several months and the maximally tolerated doses of HU may not be necessary to achieve a therapeutic effect (12). HU was subsequently approved in 2017 for the reduction in the frequency of painful crises and the need for blood transfusions in pediatric patients (>2 years) with SCD with recurrent moderate to severe painful crises.

### L-glutamine

1In 2017, L-glutamine received approval from the FDA for the treatment of SCD. L-glutamine is an amino acid required to synthesize nicotinamide adenine dinucleotide (NAD+) and its reduced form NADH. NAD+ is an important antioxidant in RBCs, so the rationale for using L-glutamine in patients with SCD is to improve the redox ratio to reduce oxidative stress, RBC sickling and hemolysis, and decrease the frequency of VOC. A multicenter, randomized, placebo-controlled, double-blind, phase 3 trial enrolled patients with sickle cell anemia (HbSS or HbSβ^0^-thalassemia) at least 5 years old at 31 sites across the United States who had a history of at least 2 pain crises during the previous year (13). This clinical trial studied the efficacy of pharmaceutical-grade L-glutamine (0.3 per kilogram of body weight per dose) administered twice daily compared to placebo in reducing the incidence of pain crises. Patients who were receiving HU at least 3 months prior to screening continued that therapy throughout the 48-week treatment period. Two hundred and thirty patients (age range, 5–58 years; 53.9% female) were randomly assigned in a 2:1 ratio to receive L-glutamine (*n* = 152) or placebo (*n* = 78). Patients receiving L-glutamine had significantly fewer pain crises than those receiving placebo (median, 3 vs. 4, *P* = 0.005) and fewer hospitalizations (2 vs. 3, *P* = 0.005). The adverse effects of low-grade nausea, non-cardiac chest pain, fatigue, and musculoskeletal pain occurred more frequently in the L-glutamine group compared to the placebo group (13).

### Crizanlizumab

Crizanlizumab is an anti-P selectin inhibitor that decreases the adhesion of white blood cells and RBCs to the endothelium that was approved by the FDA in 2019 based on a phase 2 randomized, double-blind, placebo-controlled trial. Patients with sickle cell disease (HbSS, HbSC, HbSβ^0^-thalassemia, HbSβ^+^-thalassemia, and other genotypes) who were between 16 and 65 years of age and had 2–10 sickle cell-related pain crises in the 12 months prior to enrollment received low-dose crizanlizumab (2.5 mg per kg body weight), high-dose crizanlizumab (5 mg/kg) or placebo, administered intravenously 14 times over 52 weeks. One hundred and ninety-eight patients were enrolled across 60 sites. The median rate of crises per year was significantly lower with high-dose crizanlizumab compared to placebo (median, 1.63 vs. 2.98, *P* = 0.01). The median time to first and the second crisis was significantly longer with high-dose crizanlizumab compared to placebo (first crisis, median, 4.07 vs. 1.38 months, *P* = 0.001; second crisis, median, 10.32 vs. 5.09 months, *P* = 0.02). The median rate of uncomplicated crises per year was lower with high-dose crizanlizumab compared with placebo (median, 1.08 vs. 2.91, *P* = 0.02). Adverse events such as arthralgia, diarrhea, pruritus, vomiting, and chest pain were observed (14).

### Voxelotor

Voxelotor is another DMD that was approved by the FDA in 2019. It inhibits HbS polymerization by binding covalently to the N-terminal valine of the β-globin chain in Hb to stabilize the oxygenated HbS (11). A phase 3, multicenter, double-blind, randomized, placebo-controlled trial (HOPE trial), compared the efficacy and safety of 2 dose levels of voxelotor (1500 mg and 900 mg) with placebo in patients aged 12–65 years old with SCD (HbSS, HbSC, HbSβ-thalassemia, and other variants) of different genotypes (15). Participants (*n* = 274) were randomly assigned in a 1:1:1 ratio to receive a once-daily oral dose of 1500 mg of voxelotor, 900 mg of voxelotor, or placebo. Study results showed a significantly higher percentage of study subjects with a Hb response in the 1500 mg voxelotor group (51%; 95% confidence interval [CI], 41–61) compared to the 900 mg voxelotor group (33%; 95% CI, 23–42) and placebo group (7%; 95% CI, 1–12) at 24 weeks. The annualized adjusted incidence rate of VOC was 2.77, 2.76, and 3.19 in the 1500 mg, 900 mg and placebo groups, respectively. Furthermore, there was a trend toward reduced incidence of crises over time with voxelotor compared to placebo. Most adverse events were concluded to be unrelated to voxelotor or placebo. There was no significant difference of SCD-related adverse events among the 3 groups (76% 1500 mg voxelotor, 73% 900 mg voxelotor, and 73% placebo group) (15).

### Complement inhibitors

One area of growing interest in SCD pathogenesis is complement activation, the protein-based arm of the innate immune system. The first report on complement activation in SCD was published in 1967 by Francis and Womack in which they discovered high levels of serum complement markers in patients with SCD (16). Alternative pathway (AP) of complement activation may be implicated in SCD; anaphylatoxins C3a and C5a were determined to be increased during a VOC (17, 18). In adults with SCD levels of sC5b-9, a marker of complete complement activation, was significantly elevated compared to those without SCD (19, 20). Of note is that hydroxyurea reduces the degree of complement activation: 61% of patients with SCD who did not take hydroxyurea had elevated levels of sC5b-9 compared to those on the treatment (19). To date, no complement inhibitors have been specifically FDA approved within the context of SCD nor have any large-scale clinical trials been undertaken. Nonetheless, case reports of small-scale administration of certain complement inhibitors (e.g., eculizumab and ravulizumab) within certain contexts of SCD (e.g., pregnancy, delayed hemolytic transfusion reaction, VOC) have been published (21). Further research is needed to elucidate the thorough relationship between complement and SCD.

## Allogeneic hematopoietic stem cell transplantation

A significant expertise is needed to render peri-transplant care for SCD patients. In general, pediatric patients have better outcomes. Adult patients with SCD who have acquired significant organ damage due to chronic inflammation and transplant related toxicity potentially prolonging recovery. Delayed immune reconstitution with infectious complications remain significant problems. In addition, SCD patients are at risk of rejection due to immunological responses. Blood transfusion support during the recovery period might require extensive RBC antigen matching. Targeting the bone marrow microenvironment pharmacologically or by modifying the effect of mesenchymal stromal cells might improve durable engraftment with a high degree of chimerism (22).

### Allogeneic hematopoietic stem cell transplantation from related donors

Allogeneic hematopoietic stem cell transplantation (HSCT) using matched sibling donors is the only standard curative option for SCD and offers a >90% cure rate (23). Unfortunately, <20% of patients have HLA-matched donors. In the absence of a matched sibling donor, HLA-matched unrelated donors and HLA-identical sibling cord blood (CB) donors are alternatives (24).

### Unrelated cord blood transplantation

Unrelated CB transplantation (CBT) significantly expanded the donor pool for patients with severe SCD. These transplants are associated with better outcomes and low incidences and severity of GVHD. In a study of pediatric patients, a reduced intensity regimen supported donor engraftment in the majority of patients with 100% 1 year overall survival and 78% disease-free survival (25). Historically, a major limitation associated with CBT in adults was a high rate of graft failure due to inadequate cell dose. Therefore, transplantation with double cord blood units is increasingly used in adults who lack a matched related or unrelated donor.

An *ex vivo* expanded HSC from a single umbilical cord blood unit, Omidubicel, demonstrated faster hematopoietic recovery with no differences in GVHD incidences compared to the standard myeloablative CBT (*p* < 0.001) in a phase 3 randomized clinical study (26).

### Haploidentical donor transplant

Recent data of haploidentical familial donor transplants are encouraging with high overall survival, limited toxicities, and reduced rates of acute and chronic GVHD with up to 60 months of follow-up. Expanding the donor pool to haploidentical donors such as parents, children, siblings, half-siblings, and extended family donors is a promising therapeutic option for patients with SCD. To minimize rejection and GVHD, T-cell depletion of donor grafts is performed either *ex vivo* using CD34+ positive cell selection methods or i*n vivo* with high-dose post-transplant cyclophosphamide (PTCy) (27). PTCy targeted depletion of alloreactive T cells is superior to anti-thymocyte globulin or alemtuzumab rendered non-selective T-cell depletion. Increasing total body irradiation in the preparative regimen decreased the rejection rate from 43 to 6%, with most of patients achieving full donor chimerism, and 100% survival (median follow-up 705 days) (28). Addition of thiotepa in haploidentical HSCT showed an improvement in engraftment, 20% acute GVHD rates, and 100% survival after a median follow-up of 13 months (29).

## Gene therapy

Autologous HSCT offers several advantages over allogeneic HSCT: lowering risk of serious complications, increasing donor availability, absence of GVHD and graft rejection, and strengthened rapid immune reconstitution. Granulocyte colony-stimulating factor is an effective mobilization agent but is contraindicated in SCD, because it triggers severe adverse events. The CXCR4 antagonist, plerixafor, has been successful in gene therapy trials for SCD (30, 31). However, the degree of mobilization in patients with SCD is often suboptimal, so the patient may require multiple cycles of mobilization and apheresis collection. As a pretransplant preparative regimen, RBC exchange transfusions is performed to prevent the occurrence of SCD-related morbidities associated with the HSC mobilization and procurement. Gene modification of the autologous stem cells is required to reverse the effects of HbS and sustain hematopoietic engraftment. To that end, the following include potential gene therapy avenues: HbS gene correction, hemoglobin F induction, and modified HbA gene addition. Toxicities may be associated with the conditioning chemotherapy and lentiviral vector; namely, insertional mutagenesis leading to secondary malignancies.

### Antisickling gene therapy

The first gene therapy treatment of SCD was reported in 2017 (32). LentiGlobin BB305 is a lentiviral vector that encodes a modified β-globin gene, HbA^T87Q^. HbA^T87Q^ is a modified adult hemoglobin with an amino acid substitution at position 87 from threonine to glutamine, which results in anti-sickling properties due to steric inhibition of polymerization of HbS. As of February 2021, in Group C of phase 1–2 studies, all patients (*n* = 35) who received LentiGlobin infusion engrafted at a median follow-up of 17.3 months (range 3.7 to 37.6 months) (33). The median total hemoglobin level increased from 8.5 g/dL at baseline to ≥11 g/dL from 6–36 months after infusion. HbA^T87Q^ accounted for at least 40% of total Hb and lower levels of hemolysis were observed. Severe vaso-occlusive events resolved in the 25 patients who could be evaluated who experienced a median of 3.5 events per year (range, 2–13.5) in the 24 months before enrollment. Three patients experienced vaso-occlusive events after infusion. The median time to neutrophil engraftment (absolute neutrophil count, ≥500 per microliter for 3 days) was 20 days (range, 12–35) while the median time to platelet engraftment (platelet count, ≥50 x 10^3^ per microliter for 3 days without platelet transfusion) was 36 days (range, 18–136). Adverse events such as abdominal pain, opiate withdrawal syndrome, nausea, and vomiting were reported. One case of leukopenia, one of decreased diastolic blood pressure and one of febrile neutropenia were attributed possibly or definitely to LentiGlobin infusion. No cases of hematologic malignancies were reported at 37.6 months of follow-up (33).

### Gene editing to induce fetal hemoglobin

Other gene therapy approaches being studied include upregulating the expression of HbF with CRISPR Cas-9 disruption of the BCL11a gene (34), CRISPR-Cas-12 mutation of the HGB 1 and 2 enhancer sites (35, 36), and RNAi-induced suppression of BCL11a mRNA transcription through a short hairpin RNA expressed through a lentiviral-based vector (37). Very promising results of a phase I study involving six patients suggest this is a feasible approach for HbF induction in patients with SCD. All the patients evaluated achieved robust and stable HbF induction (percentage HbF/(F+S) ranged from 20.4 to 41.3%). Clinical manifestations of SCD were reduced or absent during the 6–29 month follow-up period (37).

## Discussion

**Figure 1:**
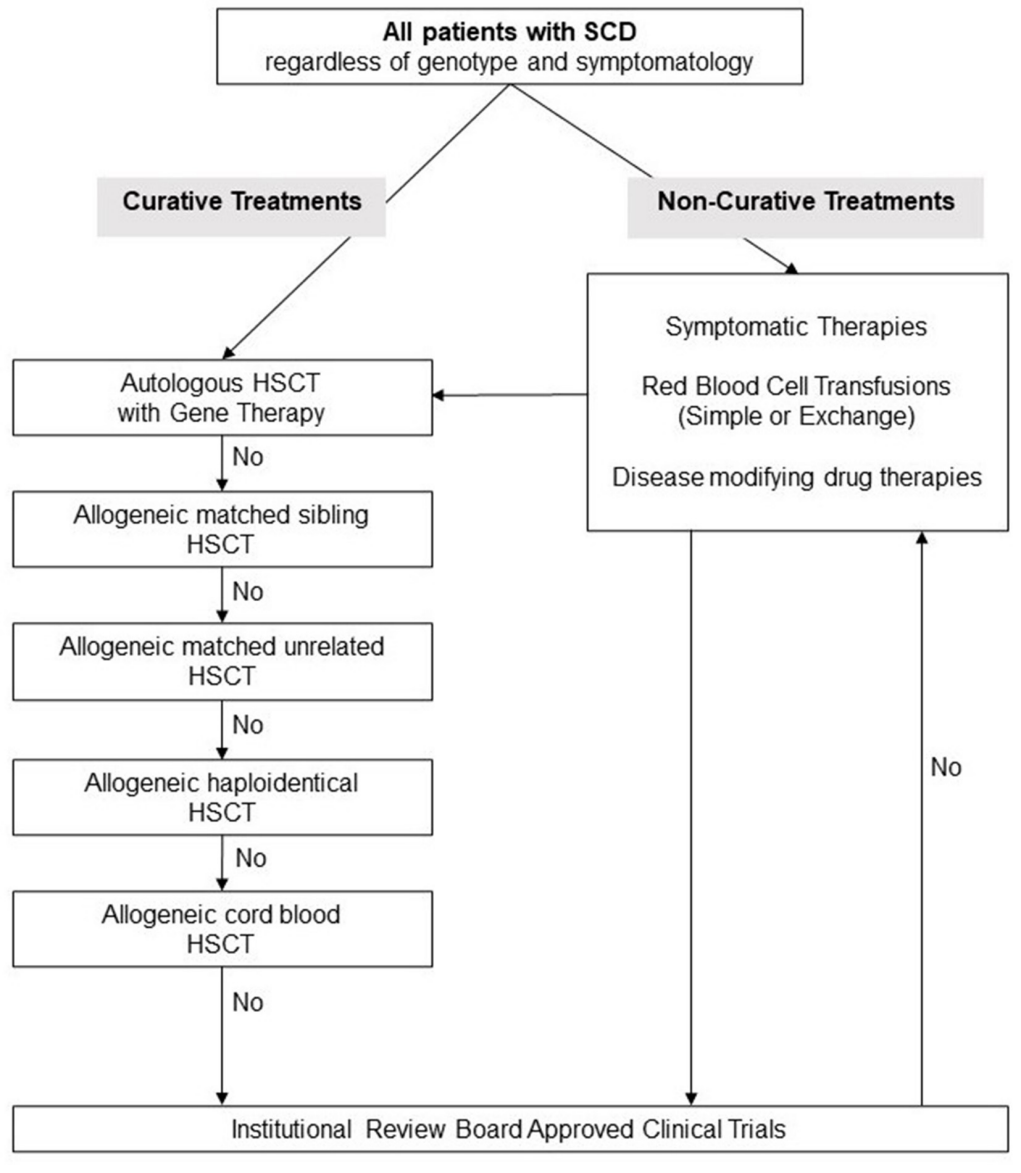
Suggested management algorithm for patients with sickle cell disease. SCD, Sickle Cell Disease; HSCT, hematopoietic stem cell transplantation.

Number of potentially curative therapies recently become available, see Figure 1 for a suggested algorithm for evaluation of patients with SCD for novel treatments. While gene therapies are promising and may perhaps cure SCD, further research is still needed before they can be utilized on all patients. Lentiviral vectors are currently preferred over gamma retroviral vectors because of their ability to transduce non-dividing primitive cells such as HPCs. A shorter *in-vitro* transduction period with lentiviral vectors is preferred in order to minimize the loss of HSC “stemness.” The long-term engraftment durability needs to be evaluated and the long-term risks of conditioning regimens such as infertility and risk of secondary malignancy must be addressed.

The high cost of transplant and gene therapy currently limits the availability of curative treatment to primarily developed countries. The estimated cost of HSCT per patient ranges from $350,000 to $800,000, while costs for gene therapy is estimated to be as high as $1- $2 million, which severely constricting access. However, this upfront cost may be acceptable when compared to the total lifetime cost of managing a patient by age 50 with a chronic disease such as SCD that exceeds $8 million (38).

Future developments of alternative therapies include pharmacologic, biologic and genetic approaches. There has been an expansion of pharmacologic agents that target mediators of inflammation in SCD, such as cellular adhesion molecules, cytokines, complement, leukotrienes, and nuclear signaling factors (39). Development of an *in vivo* gene therapy delivery system that contains nucleases packaged in viral or non-viral vehicles would bypass the need for an autologous transplant and make worldwide application logistics easier. However, reactivation of HbF using pharmacologic approaches such as small molecule regulators targeting BCL11A or other genes are more likely to be broadly implemented (40).

## Author contributions

YT, GN, and LV co-wrote the manuscript. All authors contributed to the article and approved the submitted version.

## Conflict of interest

The authors declare that the research was conducted in the absence of any commercial or financial relationships that could be construed as a potential conflict of interest.

## Publisher's note

All claims expressed in this article are solely those of the authors and do not necessarily represent those of their affiliated organizations, or those of the publisher, the editors and the reviewers. Any product that may be evaluated in this article, or claim that may be made by its manufacturer, is not guaranteed or endorsed by the publisher.
